# High levels of antimicrobial resistance at a tertiary trauma care centre of India

**Published:** 2011-03

**Authors:** Bijayini Behera, Purva Mathur

**Affiliations:** Department of Laboratory Medicine Jai Prakash Narayan Apex Trauma Centre All India Institute of Medical Sciences New Delhi 110 029, India

Sir,

Infections are a leading cause of morbidity and mortality in severely traumatized patients. The rates of infection in trauma patients are much higher than those affecting other surgical patients^1^,^2^. Since the initiation of antimicrobial therapy is often empirical, it is important to know the antimicrobial susceptibility profile of pathogens in order to select the appropriate antibiotics. We, therefore, conducted this retrospective study with the aim to review the profile of nosocomial infections and antimicrobial susceptibility of pathogens at a newly commissioned level-1 trauma care centre of India. The study was conducted at the Microbiology laboratory of Jai Prakash Narayan Apex Trauma Centre of All India Institute of Medical Sciences (AIIMS) hospital at New Delhi. The Centre has 190 beds with neurosurgery, polytrauma, general surgery and orthopaedics wards and intensive care units (ICUs) (36 bedded).

All the samples received in the Microbiology laboratory of the Trauma Centre between April 1, 2007 to March 31, 2008 were included in the study. The samples were processed according to standard microbiological techniques^3^. Antimicrobial susceptibility testing of the bacterial isolates was performed by the disk diffusion method on Mueller Hinton agar (BBL Difco, USA) according to the Clinical and Laboratory Standards Institute (CLSI) guidelines^4^. Gram-negative bacterial isolates exhibiting resistance to all available major classes of antimicrobial agents (aminoglycosides, fluoroquinolones, third generation cephalosporins, β lactams-β lactamase inhibitor combinations and carbapenems) were tested for susceptibility to polymyxin B (300 units; BBL™, BD, USA) and tigecycline (15 μg Oxoid, Basingstoke, Hants, UK; except in *Pseudomonas* and *Proteus* spp.) by the disc diffusion method. The interpretation of zone diameters was done according to the CLSI guidelines for polymyxin^5^and US FDA approved breakpoints for tigecycline^6^. Extended spectrum β-lactamase (ESBL) screening was done in all Gram negative pathogens by the double disk potentiation test using ceftazidime (Caz) (30μg) and ceftazidime + clavulanic acid (30/10 μg) (Caz+clav) disc (BBL™, BD, USA) according to the CLSI guidelines^4^. Screening for metallo β-lactamase (MBL) production was done in carbapenem resistant isolates by the imipenem- EDTA combined disc test^7^. Methicillin resistance was determined in *Staphylococcus* spp. by using oxacillin (1 μg) disc and cefoxitin (30 μg) disc methods as recommended by the CLSI^4^. The *S. aureus* strains ATCC 25923 and 43300 and *Enterococcus faecalis* ATCC 29212 strains were used as controls for sensitivity testing of Gram-positive bacteria. Extensive drug resistance was defined as an isolate resistant to all antimicrobials except one or two^8^. These included isolates resistant to carbapenems, β lactam- β lactamase inhibitor combinations, aminoglycosides, fluoroquinolones and third generation cephalosporins.

Species identification of yeast isolates was done by standard microbiological techniques^9^. For *Candida* isolates obtained from blood, antifungal susceptibility against amphotericin B and fluconazole was performed using a broth microdilution method (M27-A2) according to the CLSI guidelines^10^. Quality control was ensured by testing the CLSI recommended quality control strains *Candida parapsilopsis* ATCC 22019 (MIC range 2-8 μg/ ml) and *C. krusei* ATCC 6258 (MIC range 16-64 μg/ml). Antifungal susceptibility against amphotericin B, fluconazole, flucytosine and voriconazole was also performed by the Vitek 2 system using AST- Yst cards^11^.

During the study period, a total of 3,984 clinical specimens were received in the Microbiology laboratory. Of these, 1083 (27%) were urine samples, 890 (22%) were pus and exudates, 817 (21%) were respiratory tract specimens, 660 (17%) were blood samples, 260 (6.5%) were CVP tips/drain tube tips, 246 (6%) were body fluids from sterile sites and 28 (0.7%) were soft tissue or bone specimens. Of the 1083 urine specimens, 399 (37%) yielded growth of one/ more organisms. Similarly, 297 (36%) respiratory tract samples, 223 (34%) blood samples, 60 (24%), body fluids from sterile sites 194 (22%) pus/exudates and 36 (14%) of tips were culture positive for one or more organisms. Thus, a total of 1209 (30%) samples were culture positive.

A total of 1459 organisms were isolated from these samples. *P. aeruginosa* (321; 22%) was the most common isolate, followed by *Candida* spp. (303; 21%). Overall, Gram-negative bacilli, Gram-positive cocci and *Candida* spp. accounted for 989 (68%), 167 (11%) and 303 (21%) of the total isolates. Of the 989 Gram-negative bacteria, members of five genera (*Pseudomonas* spp., *Acinetobacter* spp., *Klebsiella* spp., *E. coli* and *Enterobacter* spp) accounted for 922 (93%) of these. ESBL production was confirmed in 802 (87%; 802/922) isolates (*Pseudomonas* spp., 292/321; 91%, *Acinetobacter* spp., 226/242; 93%, *Klebsiella* spp., 212/249; 85%, *E coli* 42/75; 56%, and *Enterobacter* spp., 30/35; 86%). *P. aeruginosa* and *A. baumannii* are the predominant nosocomial pathogens in many other hospitals across the world^12^. These are notorious nosocomial pathogens, which can survive in the hospital environment, are intrinsically resistant to many antimicrobials and commonly cause infections in the ICUs^12^. The antimicrobial susceptibility of the five most common genera in our study (*Pseudomonas* spp., *Acinetobacter* spp., *Klebsiella* spp., *E. coli* and *Enterobacter* spp.) against the five classes of antimicrobials (carbapenems, aminoglycosides, fluoroquinolones, 3^rd^ generation cephalosporins and β lactam- β lactamase inhibitor combinations) is shown in the Table. A total of 246 (27%) isolates were resistant to all these classes of drugs (extremely drug resistant). All the 246 isolates resistant to the five major classes of antimicrobials were sensitive to polymyxin. Of the 465 isolates in the five genera, which were resistant to carbapenems, MBL production was confirmed in 242 (52%) (*Pseudomonas* spp. 123, *Acinetobacter* spp. 98, *Klebsiella* spp. 18, *E. coli* 2, *Enterobacter* 1). Further, all the isolates of *E. coli, Klebsiella* spp., and *Enterobacter* spp. were sensitive to tigecycline. However, 18 (26%) of the 69 isolates of *Acinetobacter* spp., which were resistant to the five major classes of antimicrobials were also resistant to tigecycline. This is a cause for concern since tigecycline and polymyxin are being used as last resort antimicrobials in life threatening infections. A high prevalence of tigecycline resistance amongst *Acinetobacter* spp. in our study is especially worrisome since the organism is not only totally unexposed to tigecycline but also to the tetracycline group of antibiotics in our hospital.

**Table:** Resistance of Gram-negative microorganisms against five major classes of antimicrobials

Antimicrobials	*Pseudomonas* spp. (n=321) n^@^ (%)	*Acinetobacter* spp. (n=242) n^@^ (%)	*Klebsiella* spp. (n=249) n^@^ (%)	*E. coli* (n=75) n^@^ (%)	*Enterobacter* spp. (n= 35) n^@^ (%)	Total (n=922) n (%)

Carbapenems	215 (67)	180 (74)	59 (24)	6 (8)	5 (14)	465 (50)
Aminoglycosides	247 (77)	143 (59)	164 (66)	43 (57)	10 (29)	607 (66)
Fluoroquinolones	268 (83)	208 (86)	179 (72)	35 (47)	11 (31)	701 (76)
Third generation cephalosporins	295 (92)	230 (95)	219 (88)	42 (56)	30 (86)	816 (88)
β lactam-β lactamese Inhibitor combinations	240 (75)	213 (88)	116 (47)	8 (11)	5 (14)	582 (63)
Resistance to above 5 classes of drugs (XDR)	142 (44)	69 (29)	26 (10)	4 (5)	5 (14)	246 (27)

Carbapenems: represented by imipenem and meropenem; aminoglycosides represented by amikacin and netilmicin; fluoroquinolones represented by ciprofloxacin and levofloxacin; β lactam/ β lactamase inhibitor combinations represented by piperacillin/tazobacatam & cefoperazone /sulbactam; third generation cephalosporins represented by cetfazidime and ceftriaxone. (For *Pseudomonas* only ceftazidime was used);

@ n: represents number of isolates resistant to both the representative agents in a class; XDR, extremely drug resistant

Gram-positive microorganisms such as *S. aureus* and coagulase-negative Staphylococci (CoNS) were most commonly isolated from bloodstream or soft tissues. Of the 89 *S. aureus* isolates, 52 (58%) were methicillin-resistant whereas 53 (85%) of CoNS were methicillin resistant. All the 15 isolates of *Enterococcus* spp. displayed high level aminoglycoside resistance. However, only 2 (13%) were vancomycin resistant (MIC> 256 μg/ml).

Amongst *Candida* spp., *C. tropicalis* was the most common species (160, 53%), followed by *C. albicans* (57; 19%). Antifungal susceptibility test, performed for blood isolates, revealed all except one isolate of *C. rugosa* (which had a fluconazole MIC >32 μg/ml) to be sensitive for all the antifungals tested. The most common source of *Candida* spp. in our study was urine samples. Development of an ICU acquired urinary tract infection (UTI) is common in critically ill, catheterized patients. This is also seen in our ICU, where most of the traumatized patients are catheterized for long periods, especially those with spinal and head injuries. Worldwide, *Candida* Spp. are amongst the most important causes of UTI in ICUs^13^. Therefore, laboratories must optimize their means of isolating *Candida* spp. from urine specimens^13^.

An alarmingly high rate of multi-resistance in the Gram-negative bacteria was found. This is especially true for carbapenems, which are being saved for resistant Gram-negative bacterial infections. Carbapenem resistance has risen steadily over last five years at our institute^14^–^16^.

To conclude, multi drug resistant Gram-negative bacteria predominated at our centre. The high prevalence of *Candida* spp. and emerging resistance to last resort antimicrobials requires a review of empiric antimicrobial prescribing policies and strict implementation of infection control procedures.
